# Characterization of ‘*Candidatus* Syngnamydia salmonis’ (*Chlamydiales*, *Simkaniaceae*), a bacterium associated with epitheliocystis in Atlantic salmon (*Salmo salar* L.)

**DOI:** 10.1007/s00203-014-1038-3

**Published:** 2014-10-08

**Authors:** Stian Nylund, Andreas Steigen, Egil Karlsbakk, Heidrun Plarre, Linda Andersen, Marius Karlsen, Kuninori Watanabe, Are Nylund

**Affiliations:** 1 Department of Biology, University of Bergen, P.O. Box 7803, 5020 Bergen, Norway; 2 Institute of Marine Research, P.O. Box 1870, 5817 Nordnes Bergen, Norway

**Keywords:** Epitheliocystis, Chlamydia, *Simkaniaceae*, Syngnamydiae, Atlantic salmon

## Abstract

Two *Chlamydiales* have previously been found to infect Atlantic salmon (*Salmo salar* L.), *Candidatus* Piscichlamydia salmonis and *Candidatus* Clavichlamydia salmonicola. Both develop intracellularly in cyst-like inclusions in gill cells, generally referred to as epitheliocysts. Here, we present evidence for the association of a novel species of *Chlamydiales* with epitheliocystis in Atlantic salmon. Based on its partial 16S rRNA gene sequence, it is a new member of the family *Simkaniaceae*, and a 95.7 % identity to the type species *Candidatus* Syngnamydia venezia suggests inclusion in the candidate genus Syngnamydia. The presence of the bacterium in epitheliocysts in gills of Atlantic salmon was demonstrated by RNA–RNA hybridization. Ultrastructurally, the novel bacterium produces pleomorphic reticulate bodies and elementary bodies (EBs) with a characteristic morphology. The EBs are short rods with a terminal disc-like cap area, a sub-apical spherical vacuole-like electron-lucent structure and a post-equatorial nucleoid. We propose the name *Candidatus* Syngnamydia salmonis for this new agent from epitheliocysts in seawater-reared salmon .

## Introduction

Hoffman coined the term epitheliocystis (Hoffman et al. 1969) for cyst-like epidermal lesions in fish caused by bacteria ultrastructurally resembling chlamydiae (as *Bedsonia*-like = *Chlamydia*-like; *Bedsonia* a synonym of *Chlamydia*). The bacteria occur intracellularly in cyst-like inclusions; usually resulting in significantly enlarged infected cells (epitheliocystis cells). Additional evidence for a chlamydial aetiology of epitheliocystis in various fish species came from further ultrastructural observations, including documentation of a two-stage replication cycle including vegetative reticulate bodies (RBs) and infectious elementary bodies (EBs; Paperna et al. 1981; Nylund et al. 1998). Genetic studies of these agents of epitheliocystis have confirmed that they usually are members of the order *Chlamydiales* and established that the genetic diversity among them is large (Draghi et al. 2004; Meijer et al. 2006; Draghi et al. 2007; Karlsen et al. 2008; Polkinghorne et al. 2010; Camus et al. 2013; Steigen et al. 2013; Stride et al. 2013a, b). Two *Chlamydiales* have previously been found to infect and produce epitheliocyst in Atlantic salmon (*Salmo salar*), ‘*Candidatus* Piscichlamydia salmonis’ and ‘*Candidatus* Clavichlamydia^1^ salmonicola’ (Draghi et al. 2004; Karlsen et al. 2008). However, a betaproteobacterium, *Candidatus* Branchiomonas cysticola, has also recently been detected in cysts in the gills of Atlantic salmon (Toenshoff et al. 2012; Mitchell et al. 2013).

*Chlamydiae* are difficult to culture in vitro and knowledge on the genetic diversity within the phylum mainly relies on 16S rRNA gene sequences. A 16S rRNA gene-based system for classification of *Chlamydiales* has been recommended that suggests percentage sequence identity limits for the classification into taxa (Everett et al. 1999). Using these thresholds for classification, nine families have been proposed (Horn 2008, 2011; Lagkouvardos et al. 2014). Sequence data that exist from other uncultivable *Chlamydiales* from fish do, however, suggest an even higher diversity at family level (Horn 2008; Polkinghorne et al. 2010; Corsaro and Work 2012; Camus et al. 2013; Steigen et al. 2013; Stride et al. 2013a, b). The three *Chlamydiae* associated with epitheliocystis in salmonids, *Candidatus* Piscichlamydia salmonis, *Candidatus* Clavichlamydia salmonicola, and *Neochlamydia* sp. represent three different families (Horn 2008).

During the autumn of 2006, we investigated Atlantic salmon from a farm in Western Norway where the fish showed signs of respiratory distress and had prominent gill lesions. PCR testing and sequencing revealed that a suite of infectious agents were present on the gills of these salmon, including a novel epitheliocystis associated chlamydia with affinities to the family *Simkaniaceae.* The bacterium was subsequently detected in salmon from other farms in Norway.

Here, we present morphological and genetic data describing the novel chlamydia and demonstrate that its RNA is present in epitheliocysts in infected gills. We suggest a new provisional taxon, ‘*Candidatus* Syngnamydia salmonis’ in the family *Simkaniaceae*, for this new parasitic bacterium infecting Atlantic salmon.

## Materials and methods

### Material

Salmon were collected from three separate seawater farms in Norway (populations PI–PIII) in October 2006 (Table 1). PI was from Northern Norway while PII and PIII were from Western Norway. All fish suffered from gill disease with associated mortality.

**Table 1:** Number of gill samples found positive for two chlamydia in populations I–III, using real-time RT-PCR

Population	Origin (country)	Mean weight (g)	Number of positive samples/total number of samples	
			`*Cand*. P. salmonis´	Novel chlamydia
I	Nordland	264	0/10	10/10
II	Sogn-og Fjordane	365	25/25	25/25
III	Hordaland	362	1/10	10/10

Fish from PIII solely infected by the novel Chlamydia were used for in situ hybridization

Gills were collected from all populations and subsequently processed for histology and nucleic acid extraction. Gills collected from population PIII were also used for in situ hybridization (ISH).

### RNA extraction, reverse transcription RT-PCR and real-time RT-PCR

RNA was extracted from gills and transcribed into cDNA as previously described by Devold et al. (2000). PCR was run with primers 16SIGF, 806R and 16SB1 (Draghi et al. 2004), using cDNA as template for amplification of the nearly complete 16S rRNA gene of the novel chlamydia. Real-time PCR was run as described by Hodneland and Endresen (2006) with primers and probes directed against *Candidatus* Piscichlamydia salmonis (Nylund et al. 2008) and the novel chlamydia on cDNA template. The real-time assay targeting the new chlamydia consisted of specific primers SCh-F (5′-GGGTAGCCCGATATCTTCAAAGT-3′), SCh-R (5′-CCCATGAGCCGCTCTCTCT-3′) and a TaqMan^®^ FAM™ dyed minor groove binder (MGB) probe (Fam-5′-TCCTTCGGGACCTTAC-3′-MGB).

### Sequencing and sequence analysis

Sequencing of purified PCR products and plasmids was done using an ABI Prism BigDye Terminator Cycle Sequencing Ready Reaction kit, v3.1 (Applied Biosystems, Perkin-Elmer) according to producer’s recommendations. Sequencing was done in both directions, and sequences used for phylogenetic studies originated from direct sequencing of PCR products. Sequencing was performed at the sequencing facility at the University of Bergen (http://www.seqlab.uib.no).

An alignment of 56 16s rRNA gene sequences from the phylum *Chlamydiae*, retrieved from the GenBank or obtained from the present study, was made using Vector NTI 9.0 software. The alignment included members from all families within order *Chlamydiales* and several 16S rRNA gene sequences obtained from fish gills. Phylogenetic analysis was performed using TREE-PUZZLE 5.2 (available at: http://www.tree-puzzle.de), maximum likelihood (ML). The best-fit nucleotide substitution model for the dataset was GTR+I+G, identified by Modeltest 3.6 (Posada and Crandall 1998). This model was implemented. Trees were viewed using TreeView (Page 1996).

### Cloning and in vitro transcription of DIG-labelled RNA probes

Digoxigenin-labelled RNA probes against the novel chlamydia, and *Candidatus* P. salmonis and *Candidatus* B. cysticola were made as previously described (Karlsen et al. 2008). A DNA fragment (769 bp) coding for the partial 16S rRNA gene sequence from both chlamydia was amplified using primers 16sSIGF and 806R. A DNA fragment (700 bp) from the 16s DNA gene from *Candidatus* B. cysticola was amplified using primers. PCR products from *Candidatus* B. cysticola were used to make the sense/anti-sense probes (EUGB 27F: 5′-AGAGTTTGATCMTGGCTCAB-3′), (BProto-R1: 5′-GCA TTTCACCGCTACACATGG-3′). The fragment was subsequently cloned into the PCR4-vector (Invitrogen) that carries the T7 promoter. Clones with insert in opposite directions were selected as templates for transcription of RNA in the presence of DIG-labelled dUTP (Roche) to produce DIG-labelled probes in sense and anti-sense orientations. The authenticity of the probes was verified by agarose gel electrophoresis and dot blot analysis using non-labelled RNA transcripts as template.

### Histology and transmission electron microscopy (TEM)

Gill tissue samples were fixed by immersion at 6 °C in a modified Karnovsky’s fixative where distilled water had been replaced by Ringer’s solution and 4 % (w/v) sucrose solution (Nylund et al. 1995). Before embedding in EMBED-812 (Electron Microscopy Sciences), the tissues were post-fixed in 2 % (w/v) OsO_4_. Semi- and ultra-thin sections were cut on a Reichert-Jung Ultracut E (Leica). Semithin Sects. (0.5 μm) for light microscopy were stained with toluidine blue. The ultrathin Sects. (30–40 nm) were stained for 1.5 h in 5 % (w/v) aqueous uranyl acetate solution and then stained with lead citrate.

### In situ hybridization (ISH)

ISH were performed on sections of paraffin embedded gills from salmon (PIII) solely infected with the novel chlamydia in order to attach amplified sequences to gill tissue. ISH was performed as described by Xu and Wilkinson (1999) with some adaptations from other protocols: gills were fixed in 4 % paraformaldehyde in 1× PBS.DEPC.H_2_O at 4 °C over night, washed twice in 1× PBS.DEPC.H_2_O at 4 °C and dehydrated in an ethanol series in PBS.DEPC.H_2_O; 25, 50 and 70 %. Tissues were kept in 70 % ethanol at 4 °C for 3 weeks prior to embedding in paraffin wax. Tissues were further dehydrated in a Histokinette (Leica TP 1,020, Leica microsystems); 70 % ethanol, 80 % ethanol, 2 × 96 % ethanol, 2 × 100 % ethanol, 2× Xylene and 2× molten paraffin wax (60 °C). Paraffin Sects. (8 μm) were cut on a Leica RM2255 microtome and floated on a DEPC water bath at 50 °C until creases disappeared and then collected on polylysine-covered slides. The slides were dried at 40 °C and then kept at 34 °C over night. The slides were dewaxed by immersion in Histoclear (National Diagnostics) twice for 10 min. The slides were washed twice in 100 % ethanol for 2 min and rehydrated through an ethanol series in 1× PBS.DEPC.H_2_O: 100 % twice, 75, 50 and 25 % for 5 min each. Sections were washed twice in 1× PBS.DEPC.H_2_O, then once in a 0.05 M Tris–HCl buffer, pH 7.5. Sections were overlaid with Proteinase K (Promega) for 10 min (10 μg/ml) in a buffer containing 0.05 M TrisHCl and CaCl_2_ at pH 9.5. Sections were washed with 0.05 M Tris–HCl buffer, then 1× PBS.DEPC.H_2_O, refixed for 20 min in 4 % paraformaldehyde in PBS.DEPC.H_2_O in room temperature, washed three times in 1× PBS.DEPC.H_2_O for 5 min and dehydrated 25, 50, 70 and 2 × 100 % in 1× PBS.DEPC.H_2_O and dried. Sections were covered with a preheated (65 °C) hybridization mixture containing 50 % formamide, 0.6 mg/ml yeast tRNA, 2 % blocking reagent 50 μg/ml heparin, 0.1 % Triton-X-100, 5× SSC, together with denaturized sense and anti-sense probe (heated to 80 °C for 5 min, ~900 ng/ml). Slides were incubated in a moist chamber at 65 °C over night, with paper soaked in 50 % formamide and 5× SSC. Slides were washed in 2× SSC and 25 % formamide for 30 min at 65 °C, then twice for 30 min in 2× SSC, 0.5 % SDS and 0.1 % Sarcosyl, twice for 30 min in 0.2× SSC, 0.5 % SDS and 0.1 % Sarcosyl, all at 65 °C. Slides were then washed for 2 × 15 min in PBT (PBS with 0.1 % Triton X) in room temperature and then covered with 5 % sheep serum in PBT for 30 min. Anti-digoxigenin–alkaline phosphatase were added to PBT (1:2,000) and incubated at 4 °C over night (16 h). Slides were washed two times at room temperature in PBT for 10 min, then three times for 10 min in PBT, two times in NTMT (0.1 M Tris HCl buffer, pH 9.5, 0.1 % Triton-X-100, 0.1 M NaCl, 0.05 M MgCl_2_) for 5 min. NTMT were added 4.5 μl/ml of NBT and 3.5 μl/ml BCIP and incubated in dark for 8 h. Slides were then washed in PBT twice and then in DEPC.H2O and overlaid with 70 % glycerol in 0.05 M Tris HCl buffer, pH 9.5. Sections were observed in a Leitz Aristoplan light microscope, and photographs were taken with an Olympus E-330 camera.

## Results

### Sequence analysis

A cDNA fragment was initially amplified by RT-PCR from population P-II using primers specific for 16S rDNA from members in the phylum *Chlamydiae*. The same sequence (100 % identity) was subsequently obtained from salmon in population PI, while the sequence from PIII showed three substitutions (99.8 % similarity). Blast analysis of the PI sequence (Accession no: EU326493; 1342 nt) suggested it belonged to *Chlamydiales*. The highest nucleotide identity (including indels) was obtained with members of the family *Simkaniaceae*. Among these, the highest identity (98.1 %) was with an endosymbiont of the marine invertebrate *Xenoturbella* sp. (EF177461). Another closely related sequence (97.1 %) was obtained from the gills of a marine fish, the wrasse *Symphodus melops* from Norway (KC608868). *Candidatus* Syngnamydia venezia (KC182514) show highest identity (95.7 %) among the described species that produce epitheliocysts in the gills of fish. Identity with *Candidatus* Fritschea spp. was 94.3–94.5 % (AF400484, AY140911), while the 16S rRNA gene sequence of *Simkania negevensis* (U68460), the type species of the family *Simkaniceae*, showed 91.7 % identity to the novel chlamydia. Members of the *Chlamydiaceae* (e.g. D85709, CTU73110) showed an identity between 80 and 85 % to the novel chlamydia from Atlantic salmon (Table 2).

**Table 2:** Nucleotide sequence identity of the 16S rRNA gene between the novel fish *Simkaniaceae* (accession no: EU326493, 1342 nt) and other members in this family

Species	Accession numbers	(%) Identity
Family *Simkaniaceae*		
Symbiont, *Xenoturbella*	EF177461	98.1
SM081012-5S, *S. melops*	KC608868	97.1
*Candidatus* Syngnamydia venezia	KC182514	95.7
*Candidatus* Fritschea eriococci	AY140911	94.5
*Candidatus* Fritschea bernisiae	AF400484	94.3
*Simkania negevensis*	SSU68460	91.7
Family *Chlamydiaceae*		
*Chlamydia suis*	CTU73110	84.1
*Chlamydophila abortus*	D85709	83.8

Nucleotide identity is calculated as number of identities divided by total alignment length, including in/dels. SM081012-5S is a 16S rRNA gene sequence obtained from the gills of *Symphodus melops*

**Fig. 1:**
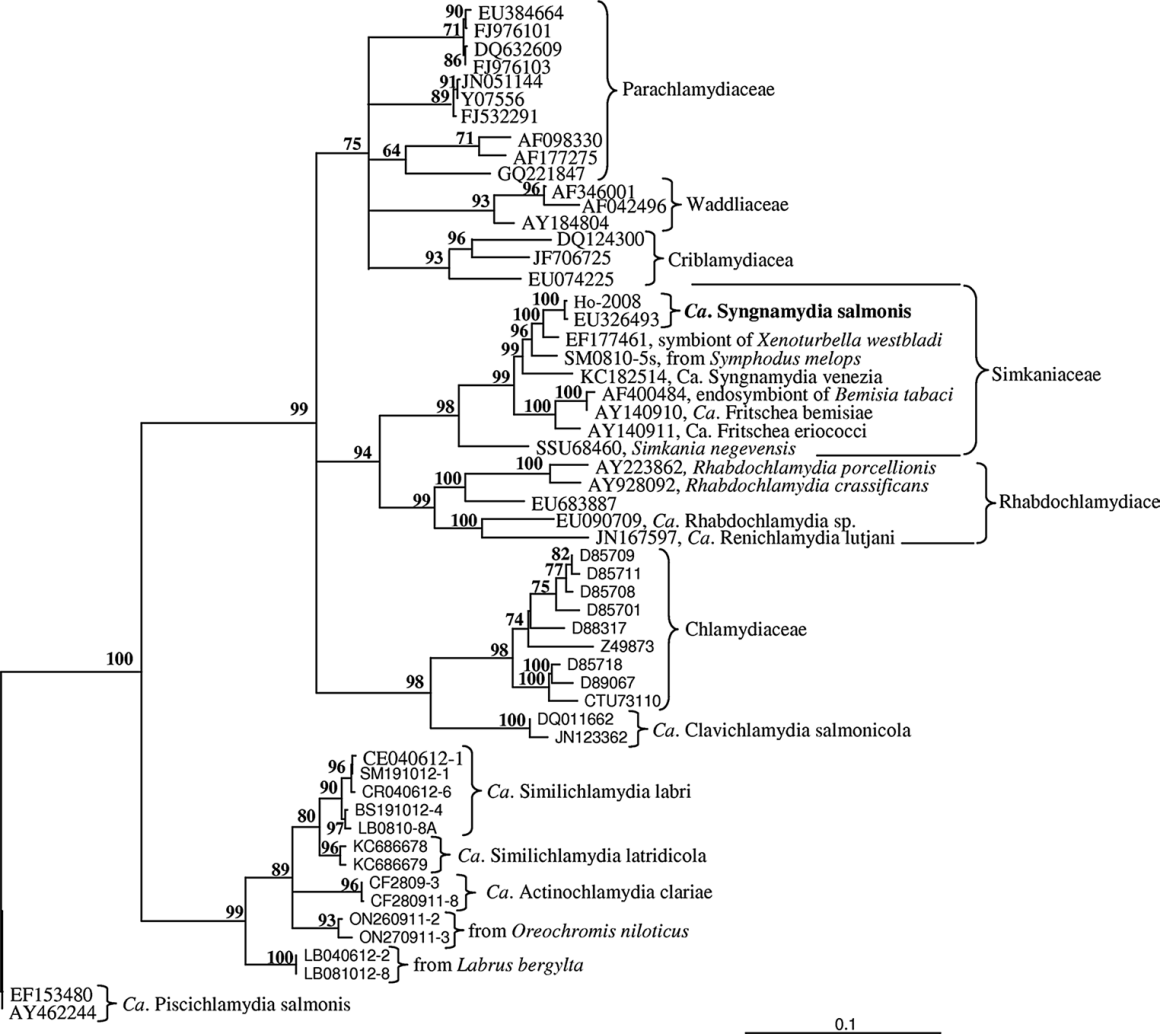
Phylogenetic position of the novel fish *Chlamydiae* (‘*Candidatus* Syngnamydia salmonis’, EU326493). The phylogeny is inferred from sequences of the 16S rRNA gene from members of the phylum *Chlamydiae*, with *Candidatus* Piscichlamydia salmonis as outgroup. Accession numbers are given in the figure. Family names from the review by Horn (2008; *Ca.* = *Candidatus)*

Phylogenetic analyses based on partial 16S rRNA gene sequences from selected members of *Chlamydiae* place the new bacterium with members of the family *Simkaniacea*. The novel chlamydia groups with the *Xenoturbella* sp. symbiont in a sub-clade together with the fish-infecting species *Candidatus* S. venezia and the unnamed species from *S. melops* (Fig. 1).

### Histology and ultrastructure of epitheliocysts

**Fig. 2:**
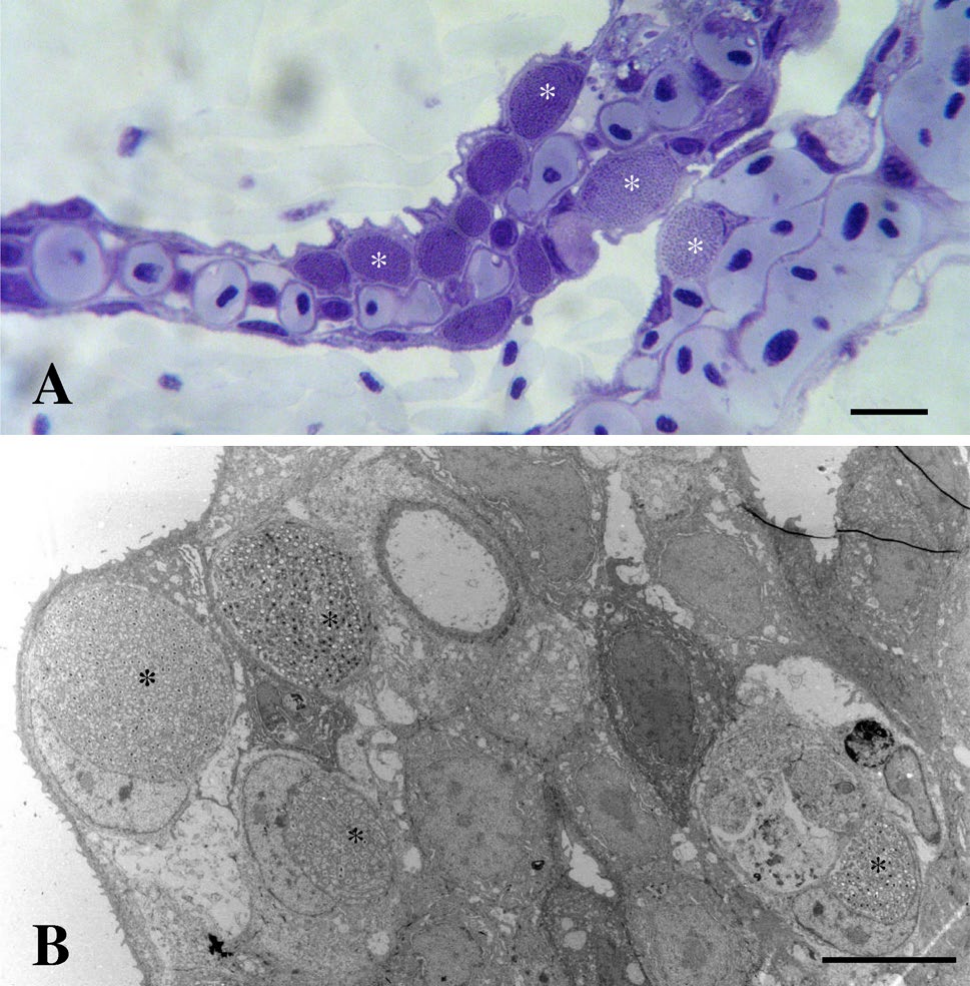
**a** Hypertrophied epithelial cells containing intracellular, intravacuolar, *Chlamydia*-like bacteria, in the gills of Atlantic salmon (*asterisks*). *Bar* 15 μm. **b** Transmission electron microscope pictures of epithelial cells infected with *Chlamydia*-like bacteria inside inclusions (*asterisks*) *Bar* 10 μm

**Fig. 3:**
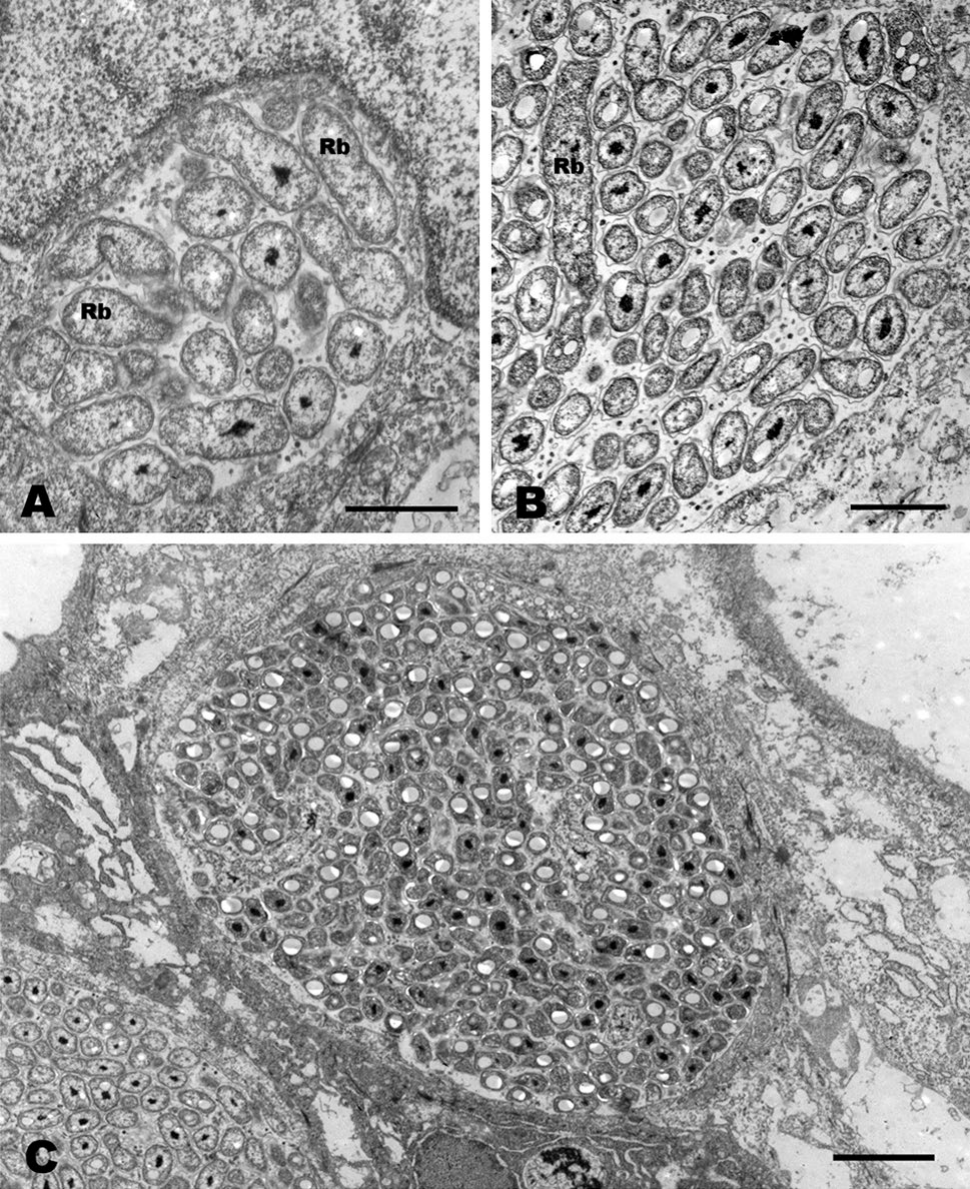
Early stages in the development of ‘*Candidatus* Syngnamydia salmonis’. **a** Infected epithelial cell containing a vacuole with reticulate bodies (*Rb*). Host cell nucleus (*Nu*). *Bar* 1 µm. **b** Epithelial cell with an intermediately sized inclusion containing a reticulate body (*Rb*) and smaller intermediate bodies with more condensed nucleoids. *Bar* 1 µm. **c** Infected epithelial cell containing mostly immature elementary bodies with distinct nucleoids and electron-lucent areas. *Bar* 2 µm

**Fig. 4:**
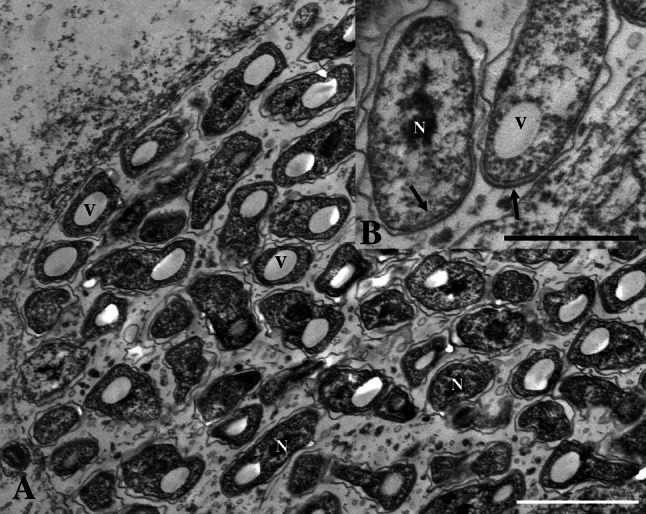
**a** Magnification of elementary bodies of ‘*Candidatus* Syngnamydia salmonis’. Nucleoid (*N*), electron-lucent area (*V*). *Bar* 1 µm. **b** Magnification of the apical cap (*arrows*) showing rod-like structures. *Bar* 0.5 µm

Histological studies of gills from all populations revealed epitheliocysts. These cysts consisted of single hypertrophied cells with an inclusion containing bacteria (Figs. 2, 3). The largest epitheliocysts had a diameter up to 25 µm. The large inclusions displaced the cell nucleus (Fig. 3a). Transmission electron microscopy revealed that in addition to bacteria, the inclusions contained small membrane bound particles, and filamentous and amorphous material (Fig. 3). The bacteria in the inclusions showed different morphologies: large elongated, branching bodies (up to 2.5 µm in length) containing granular material (ribosomes) and areas with amorphous material (chromatin), here referred to as reticulate body, RB-like (Figs. 2, 3) smaller more electron dense, short rod-like morphs (approximately 0.7–1.0 × 0.4–0.5 µm) with a distinct apical disc (cap), a sub-apical translucent vacuole-like area (average diameter of 280 nm), here referred to as likely elementary bodies (EBs) (Figs. 3, 4), morphs with an intermediate size (500–800 nm in diameter) compared to the RBs and the EBs also present in the inclusions, here referred to as intermediate bodies (IBs; Fig. 3b, c). All stages of the bacterium were surrounded by two unit membranes, in the sub-apical parts of the bacterium, the outer membrane appears irregular and detached from the inner membrane (Fig. 4). In the cap area of mature EBs, the two membranes are aligned at a fixed distance to each other. This area contains rods of electron dense material that appears to traverse both membranes and project from the cap-surface (Fig. 4a, b). In some cases, these rods are also apparent in the cytoplasm of the bacteria, reaching to the vacuole-like body. A prominent nucleoid is located centrally or slightly post-equatorially in the EBs.

### In situ RNA–RNA hybridization

**Fig. 5:**
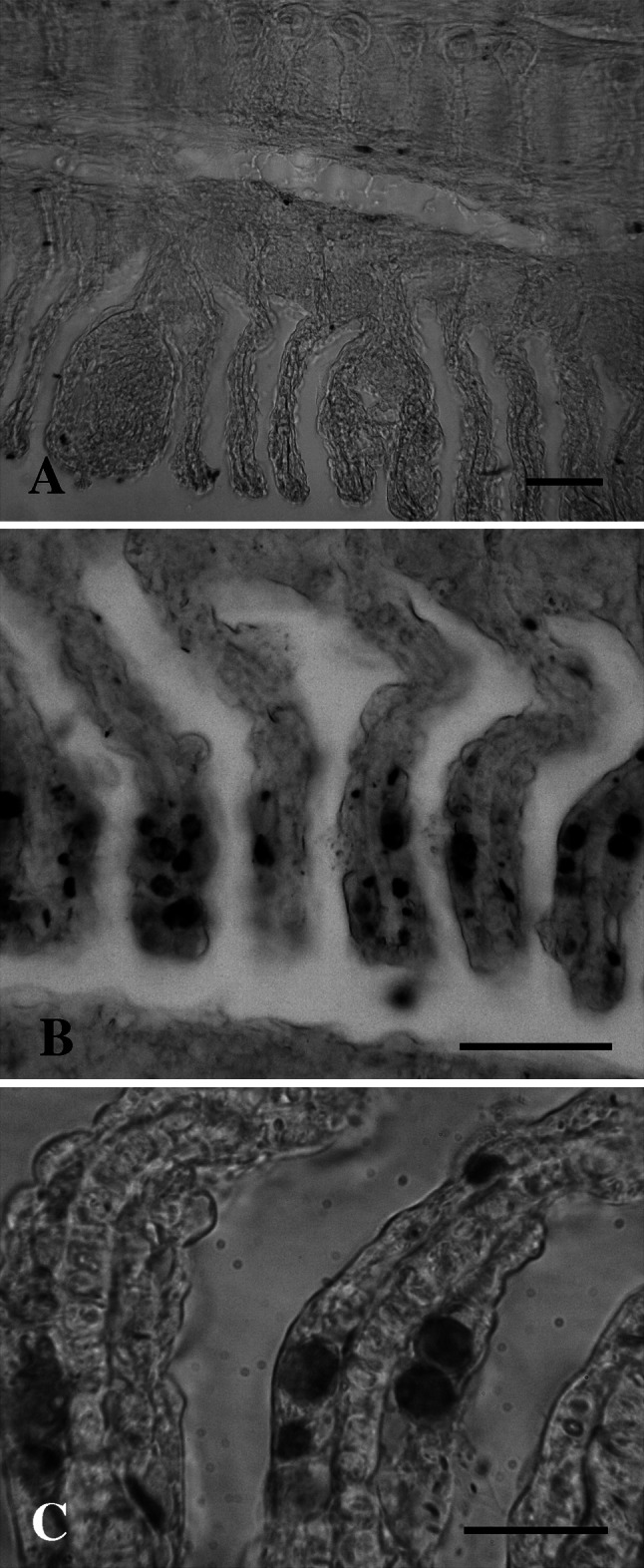
*In situ* hybridization on sections of paraffin embedded gills from ‘*Candidatus* Syngnamydia salmonis’-infected fish using DIG-labelled RNA probes. **a** Gills of infected fish stained with a sense DIG-labelled RNA probe targeting the 16S rRNA from thenovel chlamydia. *Bar* 100 µm. **b** Gills of infected fish stained with an anti-sense DIG-labelled RNA probe. Infected cells are seen as dark dots clustered together in foci. *Bar* 50 µm. **c** Higher magnification of **b** *Bar* 20 µm

In order to directly relate the amplified sequences to the epitheliocysts in the gills, sense and anti-sense DIG-labelled RNA probes were constructed from the partial 16S rDNA gene of the new *Chlamydiae* that was amplified from PI. The probes ability to hybridize to positive and negative gill tissues was tested. Gills from *S. salar* (PIII) suffering from gill diseases and positive by PCR and sequencing for the new chlamydia, reacted with the DIG-labelled anti-sense RNA probe, but not with the corresponding sense probe (Fig. 5). Stained inclusions were observed in clusters unevenly distributed on the gill lamellae but primarily located to the apical half of the secondary lamellae. They appeared always to be covered by a single layer of gill epithelium. Gills from negative *S. salar* were not stained (not shown). As additional negative controls, the homologous part of the 16S RNA gene from ‘*Candidatus* P. salmonis’ (nucleotide identity = 78.0 %) was also cloned and transcribed into sense and anti-sense DIG-labelled RNA probes. None of these probes hybridized to fragments of the infected gills P-III (not shown). The probes (sense/anti-sense) targeting the 16SrRNA from *Candidatus* B. cysticola were tested in RNA–RNA dot blots using RNA from the new chlamydia and *Candidatus* B. cysticola. These probes did not hybridize to the 16S from the new chlamydia (67 % match).

## Discussion

Gill diseases are causing significant economical losses in the salmonid fish industry in Western Norway. Several pathogens may contribute to the gill pathology observed in *S. salar*. Most of these pathogens are currently uncultivable and thus unavailable for controlled challenge experiments that could clarify their roles. Three different bacteria cause epitheliocystis in Atlantic salmon. *Candidatus* Clavichlamydia salmonicola is a freshwater species that disappears 4–6 weeks after sea transfer (Karlsen et al. 2008; Mitchell et al. 2010). *Candidatus* Piscichlamydia salmonis and *Candidatus* Branchiomonas cysticola infections are common in seawater-reared salmon, and may be abundant in cases of gill disease (Steinum et al. 2010; Nylund et al. 2011). *Candidatus* P. salmonis infections may be acquired in freshwater (Steinum et al. 2010), but infections are more severe in salmon developing PGI, suggesting a proliferation of the bacterium also during the seawater phase (Steinum et al. 2010). However, recent studies suggest that *Candidatus* P. salmonis is not a major agent of epitheliocystis in Ireland or Norway (Mitchell et al. 2013). *Candidatus* B. cysticola infections seem to be acquired in the sea (Mitchell et al. 2013).

The knowledge of the genetic diversity within the phylum *Chlamydiae* has been greatly expanded the last decade. Sequencing of environmental 16S rRNA gene clones has revealed that *Chlamydiae* are more diverse than previously assumed (Lagkouvardos et al. 2014). The *Simkaniaceae* is a relatively recent addition to the *Chlamydiae* phylum (Everett et al. 1999). The type species, *Simkania negevensis*, is a widespread human pathogen (Friedman et al. 2003; Kahane et al. 1998, 2002; Lieberman et al. 1997, 2002). Previously known members of *Simkaniacea* include insect symbionts (Everett et al. 2005), an unnamed symbiont of a marine deuterostome, *Xenoturbella* *westbladi* (Israelsson 2007; Kjeldsen et al. 2010), and *Candidatus* Syngnamydia venezia from gill epitheliocysts in the marine fish *Syngnathus typhle* (Fehr et al. 2013). Based on 16S gene sequence identity, as well as the phylogenetic analysis, the new *Chlamydiales* from Atlantic salmon gills is closest related to the chlamydia infecting *X. westbladi* (98.1 % identity), followed by *Candidatus* S. venezia (95.7 %). According to Stackebrandt and Ebers (2006), a divergence of 1.0–1.3 % in 16S rRNA gene sequences is indicative of separate bacterial species. This threshold range separates it clearly from *Candidatus* S. venezia as well as from the unnamed *Xenoturbella* chlamydial symbiont. A sequence (Accession number KC608868) from epitheliocystis infected gills of the marine labrid fish *Symphodus melops* represents an additional member of the genus (97.1 % identity). Hence, this particular lineage of *Simkaniaceae* (*Candidatus* Syngnamydia), infect very different marine host species over a wide geographical area. Indeed, the genetic relationships within the *Chlamydiae* appear not to correspond well with habitat or geographical location (Horn 2008).

The ISH experiments confirmed that the amplified sequence from the new *Chlamydiales* was present in inclusions in gill cells of Atlantic salmon. Infected cells occurred in clusters indicating that single-cell infections may have spread to neighbouring cells. The probes targeting *Candidatus* P. salmonis showed 78.0 % sequence identity to the probes targeting the new *Clamydiales* and did not react with the gill tissues positive for the latter. The betaproteobacterium *Candidatus* B. cysticola that also produce epitheliocystis in the gills of salmon (Toenshoff et al. 2012) shows a similarity of only 69.0 % with the probes used in our ISH experiment. The sense and anti-sense probes targeting the new chlamydia did not react with the 16S gene from this bacterium, nor did the probes targeting *Candidatus* B. cysticola react with the 16S gene from the new chlamydia.

The transmission electron microscopy studies of the inclusions of infected cells revealed morphotypes indicating that members of this taxon have a chlamydia-type developmental cycle. Despite that all the studied infections displayed a range of different developmental stages of *Chlamydiae,* the most structurally advanced stages seen were the presumed EBs, with a rod-armed apical cap and a characteristic large vacuole-like globule. These therefore appear to represent the mature EBs in this species. They differ from the EBs of many other epitheliocyst-forming *Chlamydiae* in being rod-shaped rather than round. EBs with similar morphology were depicted among the epitheliocystis agents studied by Nylund et al. (1998; e.g. Figs. 6A, 7, 8) from *S. salar*. These may also represent the new chlamydia. Among the closest relatives, an apical cap was observed in the chlamydial symbiont from *X. westbladi* (Israelsson 2007), but not in *Candidatus* S. venezia. Similar structures also occur in chlamydia-like bacteria from epitheliocysts in other fish hosts (Parperna and Sabnai 1980; Paperna et al. 1981; Crespo et al. 1999; Steigen et al. 2013). A structure similar to the EBs ‘vacuole’ was not observed in the intracellular chlamydia-like bacterium found in *X. westbladi* (Israelsson 2007; Kjeldsen et al. 2010) nor in *Candidatus* S. venezia. No clear EBs were observed by Fehr et al. (2013) in *Candidatus* S. venezia which may explain the lack of both a cap and perhaps also the ‘vacuole’. However, this bacterium did often contain clusters of electron-lucent regions that perhaps could represent related structures to the ‘vacuole’ found in the present study.

The present report improves our understanding of epitheliocystis and diagnosis of *Chlamydiae* infections in salmon, and it underscores that the diversity of members of *Chlamydiae* in fish is higher than previously perceived. It is noteworthy that *Chlamydiae* infecting fish do not seem to constitute any distinct phylogenetic clade but represent several lineages within this phylum. Three of the four previously known epitheliocystis agents from salmonids are members of the order *Chlamydiales* with affinities to three different families. Our study adds a fourth family, *Simkaniaceae*, to the suite of Atlantic salmon gill pathogens.

### *Description of* ‘*Candidatus* Syngnamydia salmonis’

*Candidatus* Syngnamydia salmonis; sal.mo.nis, L. n. *salmo*-*onis*, salmon; L. gen. n. *salmonis*. The provisional taxon ‘*Candidatus* Syngnamydia salmonis’ contains an intracellular bacterium that infects gill cells of *S. salar* L. (*Salmonidae*) in the marine environment. Members of this taxon show morphological features indicative of a developmental cycle of replication similar to the *Chlamydiae*.

Inclusion membrane simple and smooth. Pleomorphic reticulate bodies (RB) reach 2.5 μm in length. RBs develop into IBs 500–800 nm in length and contain a centrally located electron dense nuclear area c. 150 nm in diameter. EBs measure 0.7–1.0 × 0.4–0.5 µm. Two unit membranes surround the different developmental stages of the bacterium. EBs have a cap area, a prominent electron-lucent area and an electron dense nucleoid. The cap area is composed of a region where both membranes are aligned with a regular intermembrane space containing electron dense projections. The electron-lucent areas measure 200–350 nm in diameter.

The 16S rRNA gene of ‘*Candidatus* Syngnamydia salmonis’ has been deposited in the GenBank with Accession No. KF768762 (P–I), EU326493 (P-II) and KF768763 (P-III). `*Candidatus* Syngnamydia salmonis´ is a member of the family *Simkaniaceae*.

## Footnotes

1 First spelled Clavochlamydia by Karlsen et al. (2008), amended to Clavichlamydia in Bergey’s manual vol 4 (2010) p 866 (Horn 2011).
